# Health Behaviors

**DOI:** 10.1093/geroni/igaf122.520

**Published:** 2025-12-31

## Abstract

During this session you will gain insights into the Impact of health behaviors on Aging.

